# Association of pancreatic atrophy patterns with intraductal extension of early pancreatic ductal adenocarcinoma: a multicenter retrospective study

**DOI:** 10.1007/s00535-024-02149-0

**Published:** 2024-09-16

**Authors:** Mika Miki, Atsuhiro Masuda, Mamoru Takenaka, Hideyuki Shiomi, Takao Iemoto, Hidetaka Tsumura, Masahiro Tsujimae, Hirochika Toyama, Keitaro Sofue, Eisuke Ueshima, Shunsuke Omoto, Akihiro Yoshida, Tomohiro Fukunaga, Hidekazu Tanaka, Ryota Nakano, Shogo Ota, Takashi Kobayashi, Arata Sakai, Maki Kanzawa, Tomoo Itoh, Yuzo Kodama, SMT Study Group in Japan

**Affiliations:** 1 Division of Gastroenterology, Department of Internal Medicine, Kobe University Graduate School of Medicine, 7-5-1 Kusunoki-cho, Chuo-ku, Kobe, Hyogo 650-0017 Japan; 2 Department of Gastroenterology and Hepatology, Faculty of Medicine, Kindai University, Osakasayama, Osaka Japan; 3 Division of Gastroenterology and Hepatobiliary and Pancreatic Diseases, Department of Internal Medicine, Hyogo College of Medicine, Nishinomiya, Hyogo Japan; 4 Department of Internal Medicine, Kita-Harima Medical Center, Ono, Hyogo Japan; 5 Department of Gastroenterological Oncology, Hyogo Cancer Center, Akashi, Hyogo Japan; 6 Division of Hepato-Biliary-Pancreatic Surgery, Department of Surgery, Kobe University Graduate School of Medicine, Kobe, Hyogo Japan; 7 Department of Radiology, Kobe University Graduate School of Medicine, Kobe, Hyogo Japan; 8 Division of Diagnostic Pathology, Kobe University Graduate School of Medicine, Kobe, Hyogo Japan

**Keywords:** Focal pancreatic parenchymal atrophy, Early pancreatic cancer, Intraductal extension, Surgery

## Abstract

**Background:**

Focal pancreatic parenchymal atrophy (FPPA) and upstream pancreatic atrophy (UPA) may indicate the presence of early pancreatic cancer. In early pancreatic cancer, the tumor occasionally spreads laterally along the main pancreatic duct, presenting challenges in determining the extent of surgical resection. This study aimed to investigate the association of pancreatic atrophy pattern and intraductal cancer extension.

**Methods:**

Thirty-two patients with early-stage pancreatic cancer who underwent surgery at five participating centers were enrolled. Pancreatic atrophy was defined as the narrowing of parenchyma compared to the surrounding parenchyma and was classified as either FPPA (partial atrophy surrounding the pancreatic duct stenosis) or UPA (global atrophy caudal to the site of duct stenosis). Intraductal cancer extension was defined as an extension exceeding 10 mm.

**Results:**

Preoperative computed tomography revealed FPPA, UPA, and no parenchymal atrophy in 13, 13, and 6 patients. Cases with FPPA or UPA showed significantly longer cancer extensions than those without atrophy (*P* = 0.005 and *P* = 0.03, respectively). Intraductal cancer extension was present in all but one case of FPPA. 69% (9/13) of the cases with UPA showed intraductal cancer extension, whereas cases without atrophy showed no intraductal cancer extension. Importantly, two patients with FPPA or UPA showed positive resection margins during surgery and three patients with FPPA or UPA showed recurrence in the remnant pancreas.

**Conclusions:**

The presence of FPPA and UPA indicates lateral cancer extension in early-stage pancreatic cancer. Preoperative assessment of the pancreatic parenchyma may provide valuable insights for determining the extent of surgical resection.

**Supplementary Information:**

The online version contains supplementary material available at 10.1007/s00535-024-02149-0.

## Introduction

Advanced pancreatic cancer is a malignant tumor with a high mortality rate and poor prognosis [1]. In contrast, early pancreatic cancer, including stage 0 pancreatic carcinoma in situ (CIS), has a favorable prognosis and may be treatable with curative resection [2, 3]. However, several recent reports have shown that recurrence in the remnant pancreas can even occur in some cases of early pancreatic cancer [3–6]. Pathologically, certain cases of early pancreatic cancer, including CIS, exhibit extensive lateral cancer extension along the main pancreatic duct without invasion [7]. In such cases, special attention must be paid to the extent of surgical resection; if the margins are cancer-positive, additional resection may be required, complicating the surgical procedure, and lateral cancer extension may also be associated with a high risk of recurrence in the remnant pancreas. Therefore, preoperative assessment of intraductal cancer extension along the main pancreatic duct is necessary to determine the extent of surgical resection. However, definitive tools for predicting cancer extension are still unavailable.

Recent studies have suggested that the imaging findings in early-stage pancreatic cancer include changes in the volume of the pancreatic parenchyma [8]. Two different types of parenchymal changes have been identified: global atrophy, termed upstream pancreatic atrophy (UPA), which occurs caudal to the site of pancreatic duct stenosis, and partial atrophy, termed focal pancreatic parenchymal atrophy (FPPA), which appears surrounding the duct stenosis [8, 9]. In particular, FPPA has been recognized as a finding suggestive of early pancreatic cancer including CIS [10, 11]. Additionally, UPA is usually found in early pancreatic cancer without mass formation [9, 12]. Therefore, pancreatic atrophy, such as FPPA and UPA, can be considered an imaging finding indicating suspicion for the presence of early-stage pancreatic cancer.

Some patients with invasive pancreatic ductal carcinoma show intraductal cancer extension along the main pancreatic duct outward from the invasive area [13]. A recent study reported that cases of advanced pancreatic cancer with FPPA in previous imaging studies showed greater extension along the pancreatic duct [14]. However, in early-stage pancreatic cancer, mainly because of the limited number of cases, a comprehensive pathological evaluation of the correlation between cancer location and pancreatic atrophy has not been adequately explored. Therefore, in this study, we investigated the relationship between the pattern of pancreatic parenchymal atrophy and intraductal cancer extension using a substantial number of resected specimens of early pancreatic cancer, which is characterized by an exceptionally low diagnostic rate.

## Methods

### Study design and patients

**Fig. 1:**
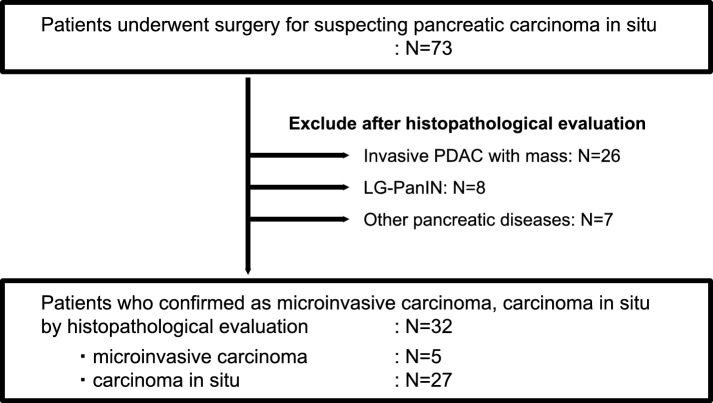
Flowchart of the study population

Between April 2013 and June 2023, 73 patients underwent surgery for suspected CIS at five participating centers: Kobe University, Kindai University, Hyogo Medical University, Kita-Harima Medical Center, and Hyogo Cancer Center. On the basis of the final diagnosis after surgery, after excluding 26 patients with invasive carcinoma presenting with mass formation, eight patients with low-grade PanIN (LG-PanIN), and seven patients with other pancreatic diseases, 32 patients were enrolled in this study. Of the 32 patients, 27 had CIS and five had microinvasive carcinoma **(**Fig. 1**)**.

Indirect findings of intraepithelial pancreatic cancer were evaluated as follows: stenosis of the main pancreatic duct with upstream duct dilatation, atrophy of the pancreatic parenchyma on computed tomography (CT), small hypoechoic areas around the ductal stenosis on endoscopic ultrasonography (EUS), and diffusion reduction areas on magnetic resonance imaging (MRI) [10, 15].

The following information was collected retrospectively from medical records: age, sex, alcohol consumption history, smoking history, family history, presence or absence of diabetes, trigger for the diagnosis, serum pancreatic enzyme levels, location of pancreatic cancer, CT findings, MRI findings, EUS findings, endoscopic retrograde pancreatography (ERP) findings, preoperative cytodiagnosis, surgical procedure, final pathological diagnosis, and clinical prognosis. We investigated the relationship between each imaging finding and the frequency and characteristics of cancer, as well as the relationship between the intraductal extension of cancer and the extent of atrophy, by comparing preoperative imaging findings with postoperative pathological findings.

All authors had access to the study data. The research protocol was prepared in accordance with the guidelines of the Declaration of Helsinki and was approved by the ethics committees of the respective centers. The study protocol was approved by the ethics committee of each institution (B220091). Eligible patients provided consent for this study in an opt-out format.

### Image evaluation

**Fig. 2:**
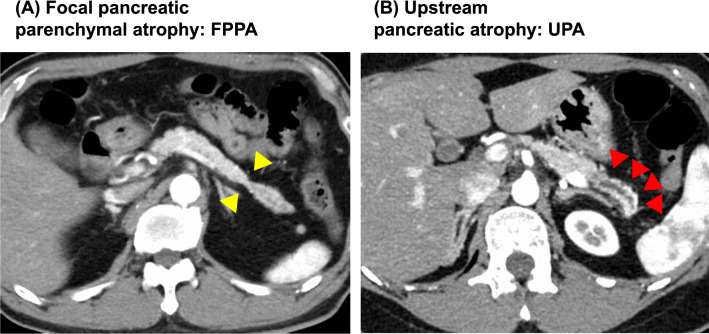
Typical CT images of atrophy of the pancreatic parenchyma in cases of carcinoma in situ. **A** Focal pancreatic parenchymal atrophy (FPPA) in the pancreatic tail (yellow arrows). **B** Upstream pancreatic atrophy (UPA) in the pancreatic tail (red arrows)

Pancreatic parenchymal atrophy is defined as narrowing of the parenchyma below a line connecting the cephalic and caudal margins of the lesion on CT images. When the pancreatic parenchyma showed “partial” atrophy such as a slit, slimness, or obvious cave-in appearance according to the criteria reported by Nakahodo et al. [8], it was referred to as FPPA, and when the upstream pancreatic parenchyma showed “global” atrophy caudal to the site of pancreatic duct stenosis in CT findings, it was referred to as UPA. Typical images are shown in Fig. 2. The changes in the diameter of the main pancreatic duct were defined as localized narrowing or irregularity of the caliber on endoscopic retrograde cholangiopancreatography (ERCP)/magnetic resonance cholangiopancreatography (MRCP). Hypoechoic areas around the main pancreatic duct stenosis on EUS were defined as borderless and coarse hypoechoic areas surrounding the main pancreatic duct stenosis. Diffusion reduction of pancreatic duct stenosis on MRI was defined as an area of high signal intensity on diffusion-weighted images consistent with the main pancreatic duct stenosis.

The imaging files from other institutions were sent to Kobe University and evaluated centrally. All images were independently evaluated by a radiologist (E.U., with 15 years of clinical experience in pancreatic imaging) and a gastroenterologist specializing in pancreatic imaging (A.M., with 23 years of clinical experience in pancreatic imaging), who were blinded to all clinical information, including preoperative pathological diagnosis. The kappa values for pancreatic parenchymal atrophy on CT scan and diffusion reduction around the main pancreatic duct stenosis are 0.82 and 0.89, respectively, both of which were high. In cases with diagnostic discrepancies, a third senior radiologist (K.S., with 19 years of clinical experience in pancreatic imaging) was consulted and made the final determination unaware of the clinical information including preoperative pathological diagnosis. EUS images are evaluated by two experienced gastroenterologists, with a high kappa value of 0.87 for the hypoechoic area on EUS. In the case of differing readings, two gastroenterologists reconfirmed and finalized the decision together.

### Pathological evaluation

Surgically sectioned specimens were immersed in 10% buffered neutral formalin and sectioned at 5-mm intervals at each institution. After paraffin embedding, formalin-fixed paraffin-embedded (FFPE) sections were prepared and stained with hematoxylin and eosin. All histological slides were sent to Kobe University from other institutions and evaluated centrally. All slides were evaluated by a pathologist (T.I.) who was blinded to the clinical information, and then reviewed by a second pathologist (M.K.) who was blinded to the clinical information. If the two pathologists made different diagnoses, they rechecked the sections and reached a diagnosis in consensus.

Pancreatic intraepithelial neoplasia (PanIN) was defined as microscopic papillary or flattened noninvasive epithelial neoplasia arising in the pancreatic duct [16, 17]; PanIN is characterized by columnar-to-cuboidal cells with varying amounts of mucin and varying degrees of cytologic and histologic atypia [18]. The degree of cytologic and histologic atypia varies and is classified into low and high grades based on histological evaluation. High-grade PanIN (HG-PanIN), classified as PanIN-3 by the WHO in 2010, is an important lesion that progresses to invasive cancer and is referred to as CIS [19, 20]; a typical image is shown in Supplementary Fig. 1A. Microinvasive carcinoma was defined as scattered cancer cells infiltrating the adjacent tissue without mass formation, as shown in a typical case in Supplementary Fig. 1B. Patients with intraductal papillary mucinous neoplasms (IPMNs) without main pancreatic duct stenosis or overt chronic pancreatitis were excluded. Pathological atrophy was defined as severe lobular atrophy and fibrosis around the main pancreatic duct upon histological examination of hematoxylin and eosin-stained specimens [11]. The length of cancer extension along the main pancreatic duct was calculated as 5 mm per section of a surgically resected pathology specimen (5 mm wide) in which cancer was present.

### Statistical analysis

Continuous variables were evaluated using Student’s t-test or Wilcoxon rank-sum test (as appropriate) and categorical variables were evaluated using the chi-square test or Fisher's exact test (as appropriate). For all analyses, a two-sided alpha level of 0.05 was used to indicate statistical significance. All statistical analyses were performed using EZR, which is available for R. More precisely, it is a modified version of R commander designed to add statistical functions frequently used in biostatistics.

## Results

### Patient characteristics according to the pattern of parenchymal atrophy

Preoperative CT revealed that of the 32 patients, 13 had FPPA, 13 had UPA, and six showed no parenchymal atrophy. Table 1 presents the patient characteristics according to the pattern of pancreatic atrophy. No significant differences were observed in age, sex, alcohol consumption history, smoking history, or family history in relation to the pattern of pancreatic atrophy. FPPA has been suggested to be less likely in the pancreatic head [8] but the present study showed no significant difference in relation to the location of the lesion and no differences in relation to abnormal serum pancreatic enzyme levels or diagnostic triggers. With regard to the final diagnosis, CIS tended to be more common in the FPPA group, although the difference was not significant.

**Table 1:** Patient characteristics categorized by the pattern of parenchymal atrophy

Characteristics	All patients		FPPA		UPA		No atrophy		*P* value
	(*N* = 32)		(*n* = 13)		(*n* = 13)		(*n* = 6)		
Age (years), Median [range]	71	[66–76]	71	[67–76]	70	[64–75]	71	[67–77]	0.99
Sex									0.45
Male	13	(40.6%)	7	(53.8%)	4	(30.8%)	2	(33.3%)	
Female	19	(59.4%)	6	(46.2%)	9	(69.2%)	4	(66.7%)	
Tumor location									0.13
Head	6	(18.8%)	1	(7.7%)	3	(23.1%)	2	(33.3%)	
Body	16	(50.0%)	10	(76.9%)	5	(38.5%)	1	(16.7%)	
Tail	10	(31.2%)	2	(15.4%)	5	(38.5%)	3	(50.0%)	
Alcohol consumption									0.51
Current	6	(18.8%)	3	(23.1%)	1	(7.7%)	2	(33.3%)	
Past	1	(3.1%)	0	(0.0%)	1	(7.7%)	0	(0.0%)	
Absent	25	(78.1%)	10	(76.9%)	11	(84.6%)	4	(66.7%)	
Smoking status									0.75
Current	7	(21.9%)	2	(15.4%)	3	(23.1%)	2	(33.3%)	
Past	7	(21.9%)	3	(23.1%)	2	(15.4%)	2	(33.3%)	
Absent	18	(56.2%)	8	(61.5%)	8	(61.5%)	2	(33.3%)	
Family history									0.19
Present	4	(12.5%)	3	(23.1%)	0	(0.0%)	1	(16.7%)	
Absent	28	(87.5%)	10	(76.9%)	13	(100.0%)	5	(83.3%)	
Diabetes mellitus									0.59
Present	4	(12.5%)	2	(15.4%)	2	(15.4%)	0	(0.0%)	
Absent	28	(87.5%)	11	(84.6%)	11	(84.6%)	6	(100.0%)	
Exacerbation of DM									0.54
Present	3	(9.4%)	1	(7.7%)	2	(15.4%)	0	(0.0%)	
Absent	29	(90.6%)	12	(92.3%)	11	(84.6%)	6	(100.0%)	
Serum amylase									0.79
≥ 133 IU/L	9	(28.1%)	4	(30.8%)	4	(30.8%)	1	(16.7%)	
< 133 IU/L	23	(71.9%)	9	(69.2%)	9	(69.2%)	5	(83.3%)	
Serum lipase									0.60
≥ 61 IU/L	7	(21.9%)	4	(30.8%)	2	(15.4%)	1	(16.7%)	
< 61 IU/L	25	(78.1%)	9	(69.2%)	11	(84.6%)	5	(83.3%)	
Trigger for diagnosis									0.29
Accidental	14	(43.8%)	5	(38.5%)	6	(46.2%)	3	(50.0%)	
Medical checkup	3	(9.4%)	2	(15.4%)	1	(7.7%)	0	(0.0%)	
Hyperpancreatic enzyme	6	(18.8%)	2	(15.4%)	3	(23.1%)	1	(10.0%)	
Diabetes mellitus	2	(6.2%)	1	(7.7%)	1	(7.7%)	0	(0.0%)	
Abdominal pain	5	(15.6%)	3	(23.1%)	2	(15.4%)	0	(0.0%)	
Acute pancreatitis	2	(6.2%)	0	(0.0%)	0	(0.0%)	2	(33.3%)	
Final diagnosis									0.14
Microinvasive carcinoma	5	(15.6%)	1	(7.7%)	4	(30.8%)	0	(0.0%)	
CIS	27	(84.4%)	12	(92.3%)	9	(69.2%)	6	(100.0%)	

(%) indicates the percentage of cases showing specific features and clinical characteristics according to the pancreatic atrophy pattern

*CIS* carcinoma in situ; *DM* diabetes mellitus; *FPPA* focal pancreatic parenchymal atrophy; *UPA* upstream pancreatic atrophy

On the other hand, when classified by pathological diagnosis, the study cohort included five cases of microinvasive carcinoma and 27 cases of CIS. The patient backgrounds for each of these groups are presented in Supplementary Table 1 and the groups showed no significant differences in the percentages of each background variable.

### Preoperative cytodiagnosis rate and waiting period

In 29 of the 32 cases, pancreatic duct brushing cytology and/or pancreatic juice cytology via ERCP were performed. The cytological diagnosis results are presented in Supplementary Table 2 and are categorized as follows: 1, benign; 2, atypical; 3, indeterminate; 4, suspicious for malignancy; and 5, malignant. In 58.6% (17/29) of these cases, the result was “suspicious for malignancy” or higher, allowing for a preoperative diagnosis. The period from diagnosis or examination to surgery ranged from a minimum of 14 days to a maximum of 169 days, with a median of 49 days.

### Relationship between the pattern of the pancreatic parenchymal atrophy and intraductal extension of early pancreatic cancer

**Fig. 3:**
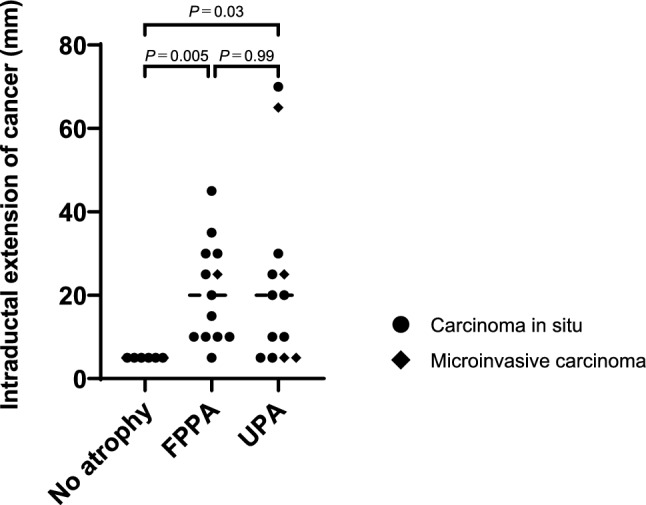
The length of intraductal cancer extension in the FPPA group, the UPA group, and the no atrophy group. The median distance of cancer extension in the FPPA, UPA, and no atrophy groups was 20.0 mm, 20.0 mm, and 5.0 mm, respectively, and both FPPA and UPA groups showed significantly longer extension than the no atrophy group

The length of intraductal cancer extension was analyzed in the FPPA, UPA, and no atrophy groups (Fig. 3). The lateral distance of cancer extension was significantly longer in cases with FPPA than in those without atrophy (median: 20.0 mm vs. 5.0 mm, *P* = 0.005). Furthermore, a significant difference was observed in the distance of cancer extension between the UPA group and the group without atrophy (median: 20.0 mm vs. 5.0 mm, *P* = 0.03).

**Fig. 4:**
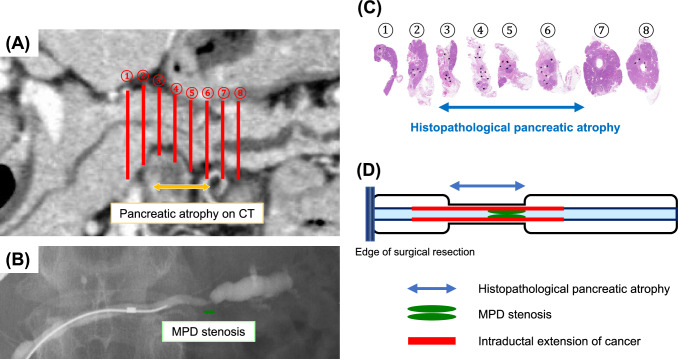
Comparison of preoperative imaging findings and postoperative pathological findings. **A** Pancreatic parenchymal atrophy was assessed using preoperative CT images by matching the location of the resected specimen. **B** The length of main pancreatic duct stenosis was evaluated by ERCP/MRCP images. **C** Pathological sections of the resected specimens were also evaluated to determine the extent of the main pancreatic duct changes and parenchymal atrophy in terms of their length and location. **D** Using a schematic diagram of the pancreas as a rectangular background with the pancreatic duct running through the center, pancreatic parenchymal atrophy on CT images is represented as a concavity, and the edge of surgical resection is indicated by a double line. The extent of histopathological pancreatic atrophy is marked with a blue arrow. The relationship between parenchymal atrophy and the location of the main pancreatic duct stenosis (depicted by a green double long circle) and the extent of intraductal cancer extension (shown by red bar lines) along the axis of the main duct was assessed

Preoperative imaging and postoperative pathological findings were compared as follows: pancreatic parenchymal atrophy was evaluated using preoperative CT images (Fig. 4A). The length of the main pancreatic duct stenosis was evaluated using ERCP/MRCP (Fig. 4B). Pathological sections of the resected specimens were also evaluated to determine the extent of the main pancreatic duct changes and parenchymal atrophy in terms of their length and location (Fig. 4C). The distance of intraductal cancer extension along the main pancreatic duct was also added, and the extent of each was examined side-by-side (Fig. 4D).

**Fig. 5:**
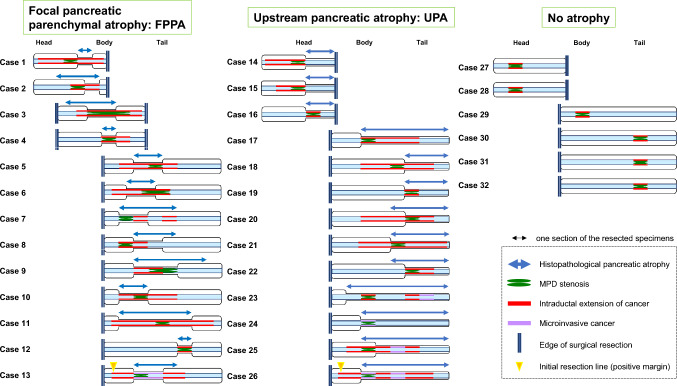
Schematic diagram of the relationship between different patterns of pancreatic parenchymal atrophy and intraductal cancer extension

A schematic diagram of the association between the pattern of pancreatic parenchymal atrophy and intraductal cancer extension is shown in Fig. 5. Among the cases with FPPA, all but one (case 12 in Fig. 5) showed longer intraductal cancer extension (≥ 10 mm, tumor located at two or more sections of the resection specimen). Cases with UPA showed two patterns of cancer extension. Among the 13 cases of carcinoma with UPA, four showed short intraductal cancer extension (< 5 mm, tumor located within one section of the resection specimen) and nine showed long intraductal cancer extension (≥ 10 mm). As for the cases without atrophy, all six cases showed short cancer extension (< 5 mm) and carcinomas located in the main pancreatic duct stenosis.

During surgery, two cases with FPPA or UPA showed positive resection margins (cases 13 and 26 in Fig. 5). Three cases with FPPA or UPA showed recurrence in the remnant pancreas after surgery (cases 10, 13, and 17 in Fig. 5), and the median follow-up periods were 44, 22, and 23.5 months in the FPPA, UPA, and no atrophy groups, respectively, with no significant differences (*P* = 0.24). And there was also no correlation between the presence or absence of post-operative recurrence and the waiting period to surgery (median time to surgery: 38 days for those with recurrence vs. 49 days for those without recurrence, *P* = 0.48). In addition, preoperative ERCP was performed in 29 of 32 cases, but not in all cases. Of the 29 cases where ERCP was performed, positive intraoperative resection margins were found in two cases and remnant pancreatic recurrence in three cases, while in the three cases where ERCP was not performed, there were no positive resection margins or remnant pancreatic recurrence. There was no association between whether ERCP was performed and positive resection margins or remnant pancreatic recurrence (*P* = 0.99).

### Frequency of each suspicious imaging finding in the early stages of PDAC

The frequencies of each suspicious imaging finding in the early stages of PDAC are shown in Table 2. All patients showed stenosis and changes in the diameter of the main pancreatic duct on ERCP or MRCP. Pancreatic parenchymal atrophy was the second-most common imaging finding after pancreatic stenosis (100% of microinvasive cancer, 78% of CIS). FPPA was observed in 12 of 27 CIS patients (44%) and in 1 of 5 patients (20%) with microinvasive cancer. On the other hand, UPA was observed only in 9 of 27 CIS patients (33%), and in 4 of 5 patients (80%) with microinvasive cancer. Hypoechoic areas around the main pancreatic duct stenosis on EUS were observed in 13 of 27 CIS patients (48%). Diffusion reduction around the pancreatic duct stenosis on MRI was also observed in 5 of 27 CIS patients (19%).

**Table 2:** Frequency of each suspicious imaging finding in the early stage of PDAC

	Microinvasive Carcinoma (*n* = 5)	Carcinoma in situ (*n* = 27)
MPD stenosis	100% (5/5)	100% (27/27)
Any pancreatic parenchymal atrophy on CT scan	100% (5/5)	78% (21/27)
Focal parenchymal atrophy	20% (1/5)	44% (12/27)
Upstream pancreatic atrophy	80% (4/5)	33% (9/27)
Hypoechoic areas around the MPD stenosis on EUS	40% (2/5)	48% (13/27)
Diffusion reduction around the MPD stenosis on MRI	20% (1/5)	19% (5/27)

*CT* computed tomography; *EUS* endoscopic ultrasonography; *MPD* main pancreatic duct; *MRI* magnetic resonance imaging; *PDAC* pancreatic ductal adenocarcinoma

## Discussion

In this study, we observed a significant correlation between the pattern of pancreatic atrophy and lateral extension in early pancreatic cancer. In particular, patients with FPPA exhibited significantly longer lateral cancer extensions than those without atrophy. All but one patient with FPPA showed intraductal cancer extension. To the best of our knowledge, this is the first study to evaluate the correspondence between atrophy on preoperative CT images and pathologic cancer extension in early pancreatic cancer in detail.

Nakahodo et al. reported similar results regarding the association between FPPA and cancer extension in patients with advanced pancreatic cancer. They concluded that PDAC with FPPA on “previous” preoperative imaging showed a higher rate of HG-PanIN positivity at the resection margins [14]. On the basis of their results, the authors concluded that PDAC with FPPA involves widespread HG-PanIN and requires a wide surgical margin for surgical excision. However, most of the cases in their study involved advanced cancers, whereas our study focused only on early pancreatic cancers, including CIS and microinvasive cancers. In addition, our study differs significantly in that it used FFPE sections to evaluate the extent of cancer extension and atrophy in detail, whereas the previous study evaluated the presence or absence of cancer only at the resection margins.

As suggested by the previous study and our study, long-axis extension of HG-PanIN is more likely to result in positive surgical margins for cancer and caution should be exercised in surgeries where curative resection is planned. This study included two cases of microinvasive carcinoma in which the resection margins were positive for HG-PanIN during surgery (cases 13 and 26 in Fig. 5). In one case, a 3-mm microinvasive carcinoma with FPPA on preoperative CT images was treated with distal pancreatectomy, and the resected macroscopic specimen showed an HG-PanIN extending over seven segments. At the time of surgery, the resection margin was positive for cancer and an additional resection was performed.

Preoperative prediction of long extension of CIS is useful for determining the extent of resection during surgery. Another case of microinvasive carcinoma requiring additional surgical resection is also presented (Supplementary Fig. 2; case 26 in Fig. 5). The patient underwent distal pancreatectomy with caudal pancreatic duct dilatation and UPA and was diagnosed as showing a 3-mm microinvasive carcinoma. A macroscopic specimen showed HG-PanIN extension over 14 sections and intraoperative resection margins positive for HG-PanIN, and additional resections were performed. Interestingly, in this case, images from 3 years prior to diagnosis were available, and localized atrophy of the pancreatic body was observed once. The atrophy progressed over the years and the imaging findings changed to UPA with dilatation of the main pancreatic duct. Of the 13 cancer cases with UPA, nine showed intraductal cancer extension, while four cases showed short extension with cancer in only one segment. These results suggest that CIS with UPA can be classified into two types: the lateral extension type, which may involve the transition from FPPA to UPA with intraductal cancer extension, and the short-segment type, which involves atrophy of the caudal pancreatic parenchyma due to severe stenosis of the main pancreatic duct. In other words, patients with FPPA at diagnosis or on previous images may show lateral extension of HG-PanIN. Even in cases of UPA, intraoperative resection margins require special attention and previous images, if available, should be reviewed.

In addition, in the lateral extension type of UPA, the cancer sometimes extends both upward (cephalically) and downward (caudally) from the main pancreatic duct stenosis. In the short segment type of UPA, imaging shows a significant change in the pancreatic duct diameter above and below the stenosis. Pathologically, there is a notable difference in adenocyte density in these regions, with greater glandular atrophy observed upstream. In these "short" cases, it is possible that the severe stenosis of the main pancreatic duct caused by the cancer has led to distal dilation and pancreatic parenchymal atrophy. When comparing the change in pancreatic duct diameter (caudal/cephalic) due to stenosis, the median increase was 2.0 times in the long extension type and 3.1 times in the short segment type. Although the change was greater in the latter, the difference was not statistically significant (*P* = 0.076). In cases of UPA, relatively mild dilation of the distal main pancreatic duct may result in further extension of the cancer, warranting greater caution in the extent of resection.

FPPA is a characteristic finding of intraepithelial carcinoma; pathologically, these cases show desquamation of the adenohypophysis with fibrosis and fat replacement [8]. The mechanism underlying these findings is unknown but it has been suggested to be attributable to localized pancreatitis caused by obstruction of the branching pancreatic ducts. FPPA was found not only around HG-PanIN but also around LG-PanIN. The cases reviewed in this study included eight cases of LG-PanIN. These included one case in which localized pancreatic atrophy was observed even with LG-PanIN, but this case showed "long" lesion extension, with the equivalent of LG-PanIN2 in the old protocol extended to four sections. The presence of FPPA may indicate a long PanIN lesion.

Although the prognosis after surgical resection of pancreatic cancer has improved, the incidence of remnant pancreatic cancer after surgery has also increased. The incidence of remnant pancreatic cancer after surgery is said to range from 0.7 to 26.7% [21]. The incidence of remnant pancreatic cancer has been shown to be higher in early-stage pancreatic cancer [3, 5] and Miyasaka et al. reported that the cumulative incidence of remnant pancreatic cancer was comparable between early- and advanced-stage groups [22]. In the present study, among the 32 cases included, three showed remnant pancreatic cancer during the observation period. These included two cases of CIS and one case of microinvasive carcinoma, with HG-PanIN extension distances of 20, 20, and 25 mm, all of which showed atrophy (one UPA and two FPPA). In contrast, no recurrence was observed in patients without atrophy or intraductal cancer extension. These results suggest that in cases showing long intraductal carcinoma extension with FPPA, precancerous lesions, including LG-PanIN, may remain latent in the residual pancreas after resection and indicate a high risk of recurrence. Therefore, even in early-stage pancreatic cancer, the occurrence of remnant pancreatic cancer after surgery should be noted. A longer PanIN suggests a higher rate of postoperative residual pancreatic recurrence, which is meaningful for evaluation. Thus, in cases of FPPA, special attention may be needed not only for the extent of resection during surgery, but also for postoperative recurrence.

In summary, in cases of FPPA, the cancer is thought to be located in the area of atrophy and often extends beyond it, requiring the inclusion of the atrophic area in the surgical resection to ensure clear margins and minimize recurrence in the remnant pancreas. In cases of UPA, the cancer is centered on the main pancreatic duct stenosis, and the type of intraductal extension can be categorized into “long” extension in the longitudinal axis and “short” segment confined to the main pancreatic duct stenosis. As shown in Supplementary Fig. 2, cases with UPA at diagnosis but FPPA on previous images may show lateral cancer extension and should be referred if previous images are available. The surgical technique is determined by the location of the main pancreatic duct stenosis, but in cases where the pathological diagnosis shows “long” extension, attention should also be paid to recurrence in the remnant pancreas.

On the other hand, in our study, there were six cases of short-segment CIS without atrophy. In a study by M. Ikeda et al., it was reported that non-invasive cancer parts (PanIN-3 lesions) continuously spreading from the invasive cancer area were histologically categorized into three types: flat (F), low papillary (LP), and mixed (flat and low papillary) [13]. The LP type tends to spread horizontally along the pancreatic ducts, while the F type tends to spread vertically with minimal lateral spread along the ducts. In our study, the short-segment type without atrophy included three cases of the F type and three cases of the LP or mixed type. On the other hand, all cases of the long extension type were of the LP or mixed type. This suggests that the short-segment type without atrophy can exhibit two patterns of spread: horizontal and vertical. However, the genetic and developmental differences between the two types of pancreatic cancer, F type and LP type, remain to be elucidated. Further research is required on this issue.

Our study had several limitations. First, this was a retrospective study, and the sample size was small, with a limited number of cases from a limited number of institutions. However, in general, cases of CIS are extremely rare and this study included detailed pathological assessments of a sufficiently large number of cases. Second, the studies were limited to surgical cases to exclude pathologically undiagnosed cases. Additionally, because CIS was examined on the basis of the presence of main pancreatic duct stricture, only cases showing changes in the main pancreatic duct were included. Third, there are discrepancies in the preoperative evaluation. Not all cases underwent both ERCP and MRCP, and the results of the two tests are not entirely consistent. The assessment of changes in the diameter of the main pancreatic duct was completed using ERP findings in cases where ERCP was performed and MRCP was used instead in the three cases where ERCP was not performed. Of the 27 cases where both tests were performed, 92.6% (25/27) showed similar pancreatic duct stenosis on both tests. The concordance of findings between ERP and MRCP for each case is detailed in Supplementary Table 2. Not all MRI images were captured using the same model. Of the 30 cases in which MRI was performed, 15 were obtained using 3 T imaging, while the remaining cases were captured with 1.5 T imaging. However, findings of diffusion reduction on MRI and pancreatic duct stenosis on MRCP could also be identified under 1.5 T imaging conditions. In the comparison between MRCP and ERP for main pancreatic duct stenosis described above, the two cases in which the main pancreatic duct stenosis was difficult to assess on MRCP were imaged at 3 T.

In conclusion, HG-PanIN may extend into the pancreatic duct in CIS and microinvasive carcinomas presenting with FPPA and UPA. In such cases, caution should be exercised while determining the extent of surgical resection.

## Supplementary Information

Below is the link to the electronic supplementary material.

Supplementary file1 (PPTX 103 KB)

Supplementary file2 (DOCX 33 KB)

Supplementary file3 (XLSX 13 KB)

Supplementary file4 Supplementary Figure 1 Typical images of pathological findings. (A) High-grade PanIN is defined as a noninvasive epithelial neoplasm within the pancreatic duct that is histologically characterized by severe cytological and architectural atypia. (B) Microinvasive carcinoma is defined as scattered cancer cells that have invaded adjacent tissues without forming a mass (red arrows) (PPTX 4424 KB)

Supplementary file5 Supplementary Figure 2 CT images showing progression from FPPA to UPA in a case of microinvasive carcinoma. In the pre-diagnostic images, FPPA was once present (yellow arrow) but changed to UPA (red arrows) one year before diagnosis. In the schematic diagram of the relationship between the extent of cancer extension and atrophy, areas of microinvasion are indicated by purple bars (PPTX 810 KB)
