# Supplementary material for: Association of pancreatic atrophy patterns with intraductal extension of early pancreatic ductal adenocarcinoma: a multicenter retrospective study
Source: J Gastroenterol. 2024 Sep 16;59(12):1133–42. doi: 10.1007/s00535-024-02149-0 (PMC11541273; doi:10.1007/s00535-024-02149-0)
Supplement: Supplementary file 1 — Supplementary file1 (PPTX 103 KB) [file 535_2024_2149_MOESM1_ESM.pptx]

## Slide 1
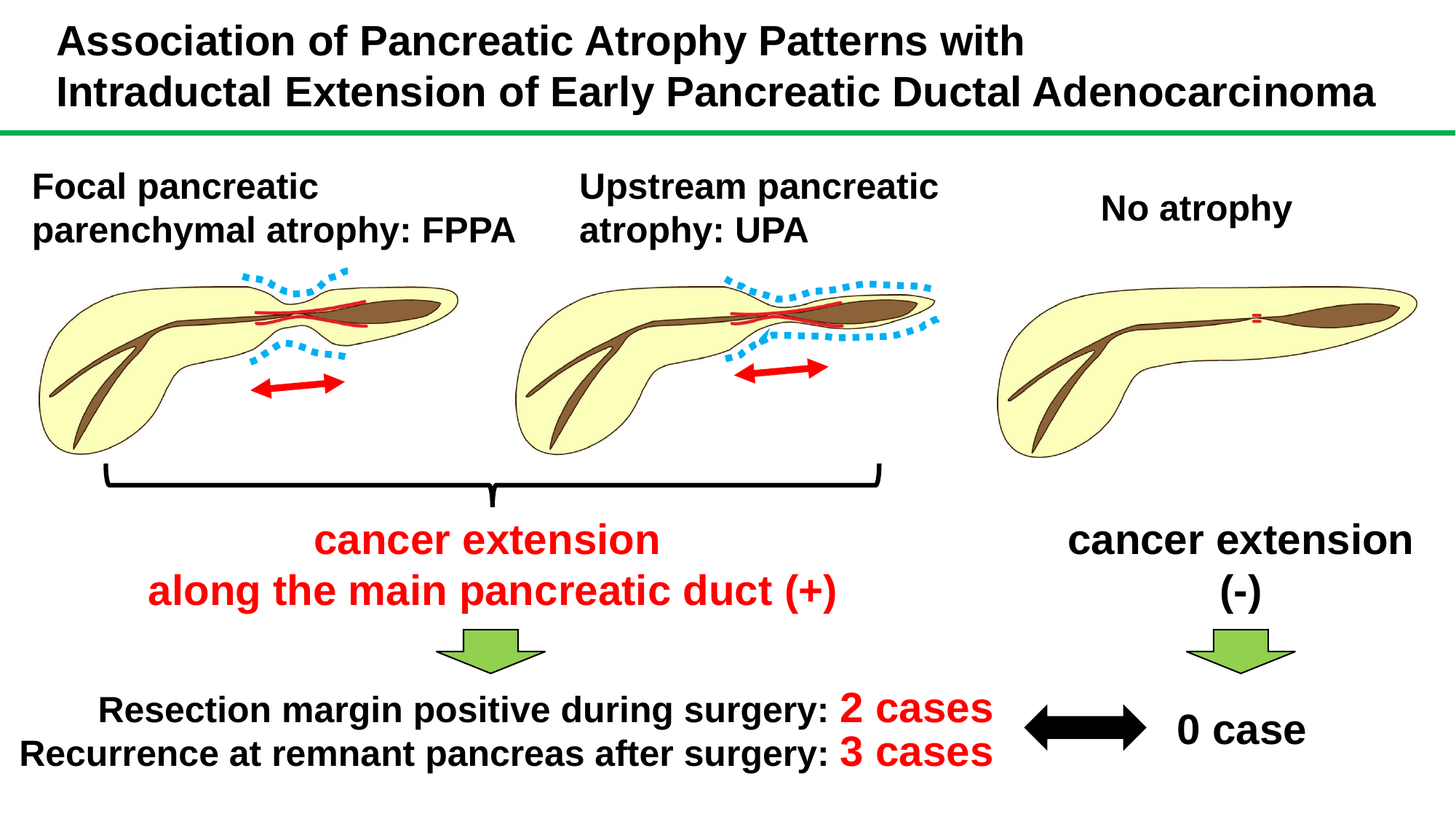

Association of Pancreatic Atrophy Patterns with
Intraductal Extension of Early Pancreatic Ductal Adenocarcinoma
Focal pancreatic parenchymal atrophy: FPPA
Upstream pancreatic
atrophy: UPA
No atrophy
cancer extension
along the main pancreatic duct (+)
cancer extension
(-)
Resection margin positive during surgery: 2 cases
0 case
Recurrence at remnant pancreas after surgery: 3 cases
