# Supplementary material for: Association of pancreatic atrophy patterns with intraductal extension of early pancreatic ductal adenocarcinoma: a multicenter retrospective study
Source: J Gastroenterol. 2024 Sep 16;59(12):1133–42. doi: 10.1007/s00535-024-02149-0 (PMC11541273; doi:10.1007/s00535-024-02149-0)
Supplement: Supplementary file 2 — Supplementary file2 (DOCX 33 KB) [file 535_2024_2149_MOESM2_ESM.docx]

**Supplementary Table 1.** **Clinical characteristics of patients according to the**

**histopathological diagnosis**

| **Characteristics** | **All patients** | | **Microinvasive**  **carcinoma** | | **Carcinoma**  **in situ** | | ***P* value** |
| --- | --- | --- | --- | --- | --- | --- | --- |
|  | **(N = 32)** | | **(n = 5)** | | **(n = 27)** | |  |
| Age (years),  Median [range] | 71 | [66-76] | 73 | [66-74] | 71 | [66-77] | 0.99 |
| Sex |  |  |  |  |  |  | 0.63 |
| Male | 13 | (40.6%) | 1 | (20.0%) | 12 | (44.4%) |  |
| Female | 19 | (59.4%) | 4 | (80.0%) | 15 | (55.6%) |  |
| Tumor location |  |  |  |  |  |  | 0.089 |
| Head | 6 | (18.8%) | 0 | (0.0%) | 6 | (22.2%) |  |
| Body | 16 | (50.0%) | 5 | (100.0%) | 11 | (40.7%) |  |
| Tail | 10 | (31.2%) | 0 | (0.0%) | 10 | (37.0%) |  |
| Alcohol consumption |  |  |  |  |  |  | 0.12 |
| Current | 6 | (18.8%) | 0 | (0.0%) | 6 | (22.2%) |  |
| Past | 1 | (3.1%) | 1 | (20.0%) | 0 | (0.0%) |  |
| Absent | 25 | (78.1%) | 4 | (80.0%) | 21 | (77.8%) |  |
| Smoking status |  |  |  |  |  |  | 0.58 |
| Current | 7 | (21.9%) | 1 | (20.0%) | 6 | (22.2%) |  |
| Past | 7 | (21.9%) | 0 | (0.0%) | 7 | (25.9%) |  |
| Absent | 18 | (56.2%) | 4 | (80.0%) | 14 | (51.9%) |  |
| Family history |  |  |  |  |  |  | 0.51 |
| Present | 4 | (12.5%) | 1 | (20.0%) | 3 | (11.1%) |  |
| Absent | 28 | (87.5%) | 4 | (80.0%) | 24 | (88.9%) |  |
| Diabetes mellitus |  |  |  |  |  |  | 0.51 |
| Present | 4 | (12.5%) | 1 | (20.0%) | 3 | (11.1%) |  |
| Absent | 28 | (87.5%) | 4 | (80.0%) | 24 | (88.9%) |  |
| Exacerbation of DM |  |  |  |  |  |  | 0.41 |
| Present | 3 | (9.4%) | 1 | (20.0%) | 2 | (7.4%) |  |
| Absent | 29 | (90.6%) | 4 | (80.0%) | 25 | (92.6%) |  |
| Serum amylase |  |  |  |  |  |  | 0.60 |
| ≥133 IU/L | 9 | (28.1%) | 2 | (40.0%) | 7 | (25.9%) |  |
| <133 IU/L | 23 | (71.9%) | 3 | (60.0%) | 20 | (74.1%) |  |
| Serum lipase |  |  |  |  |  |  | 0.56 |
| ≥61 IU/L | 7 | (21.9%) | 0 | (0.0%) | 7 | (25.9%) |  |
| <61 IU/L | 25 | (78.1%) | 5 | (100.0%) | 20 | (74.1%) |  |
| Trigger for diagnosis |  |  |  |  |  |  | 0.81 |
| Accidental | 14 | (43.8%) | 2 | (40.0%) | 12 | (44.4%) |  |
| Medical checkup | 3 | (9.4%) | 0 | (0.0%) | 3 | (11.1%) |  |
| Hyperpancreatic  enzyme | 6 | (18.8%) | 1 | (20.0%) | 5 | (18.5%) |  |
| Diabetes mellitus | 2 | (6.2%) | 0 | (0.0%) | 2 | (7.4%) |  |
| Abdominal pain | 5 | (15.6%) | 2 | (40.0%) | 3 | (11.1%) |  |
| Acute pancreatitis | 2 | (6.2%) | 0 | (0.0%) | 2 | (7.4%) |  |

(%) indicates the percentage of cases with specific features and clinical characteristics according to the histopathological diagnosis.

DM, diabetes mellitus
