# Supplementary figures and images for: Association of pancreatic atrophy patterns with intraductal extension of early pancreatic ductal adenocarcinoma: a multicenter retrospective study
Source: J Gastroenterol. 2024 Sep 16;59(12):1133–42. doi: 10.1007/s00535-024-02149-0 (PMC11541273; doi:10.1007/s00535-024-02149-0)

## Slide 1
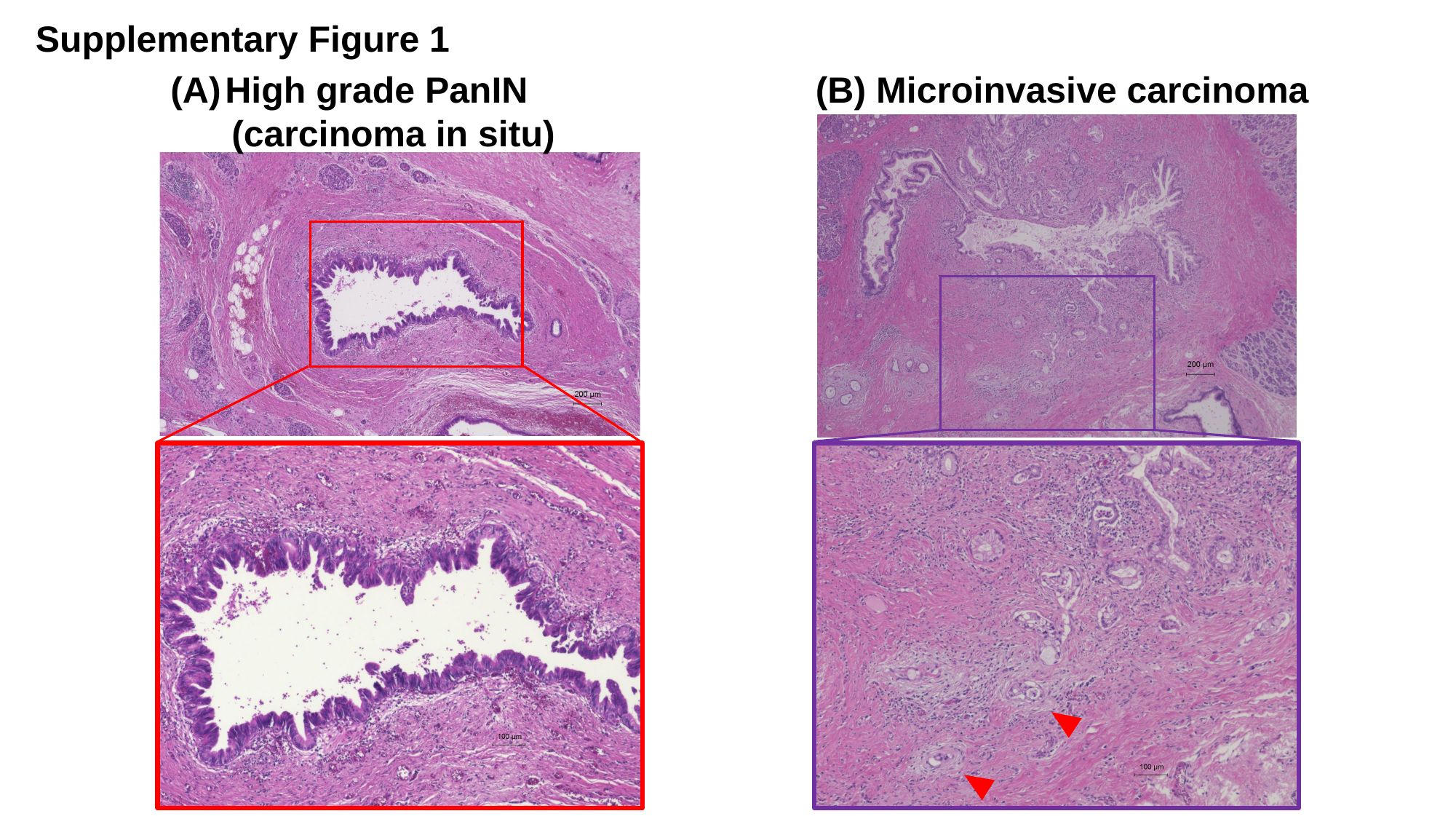

Supplementary Figure 1
High grade PanIN
 (carcinoma in situ)
(B) Microinvasive carcinoma

Supplement: Supplementary file 4 — Supplementary file4 Supplementary Figure 1 Typical images of pathological findings. (A) High-grade PanIN is defined as a noninvasive epithelial neoplasm within the pancreatic duct that is histologically characterized by severe cytological and architectural atypia. (B) Microinvasive carcinoma is defined as scattered cancer cells that have invaded adjacent tissues without forming a mass (red arrows) (PPTX 4424 KB) [file 535_2024_2149_MOESM4_ESM.pptx]
