# Supplementary material for: Association of pancreatic atrophy patterns with intraductal extension of early pancreatic ductal adenocarcinoma: a multicenter retrospective study
Source: J Gastroenterol. 2024 Sep 16;59(12):1133–42. doi: 10.1007/s00535-024-02149-0 (PMC11541273; doi:10.1007/s00535-024-02149-0)
Supplement: Supplementary file 5 — Supplementary file5 Supplementary Figure 2 CT images showing progression from FPPA to UPA in a case of microinvasive carcinoma. In the pre-diagnostic images, FPPA was once present (yellow arrow) but changed to UPA (red arrows) one year before diagnosis. In the schematic diagram of the relationship between the extent of cancer extension and atrophy, areas of microinvasion are indicated by purple bars (PPTX 810 KB) [file 535_2024_2149_MOESM5_ESM.pptx]

## Slide 1
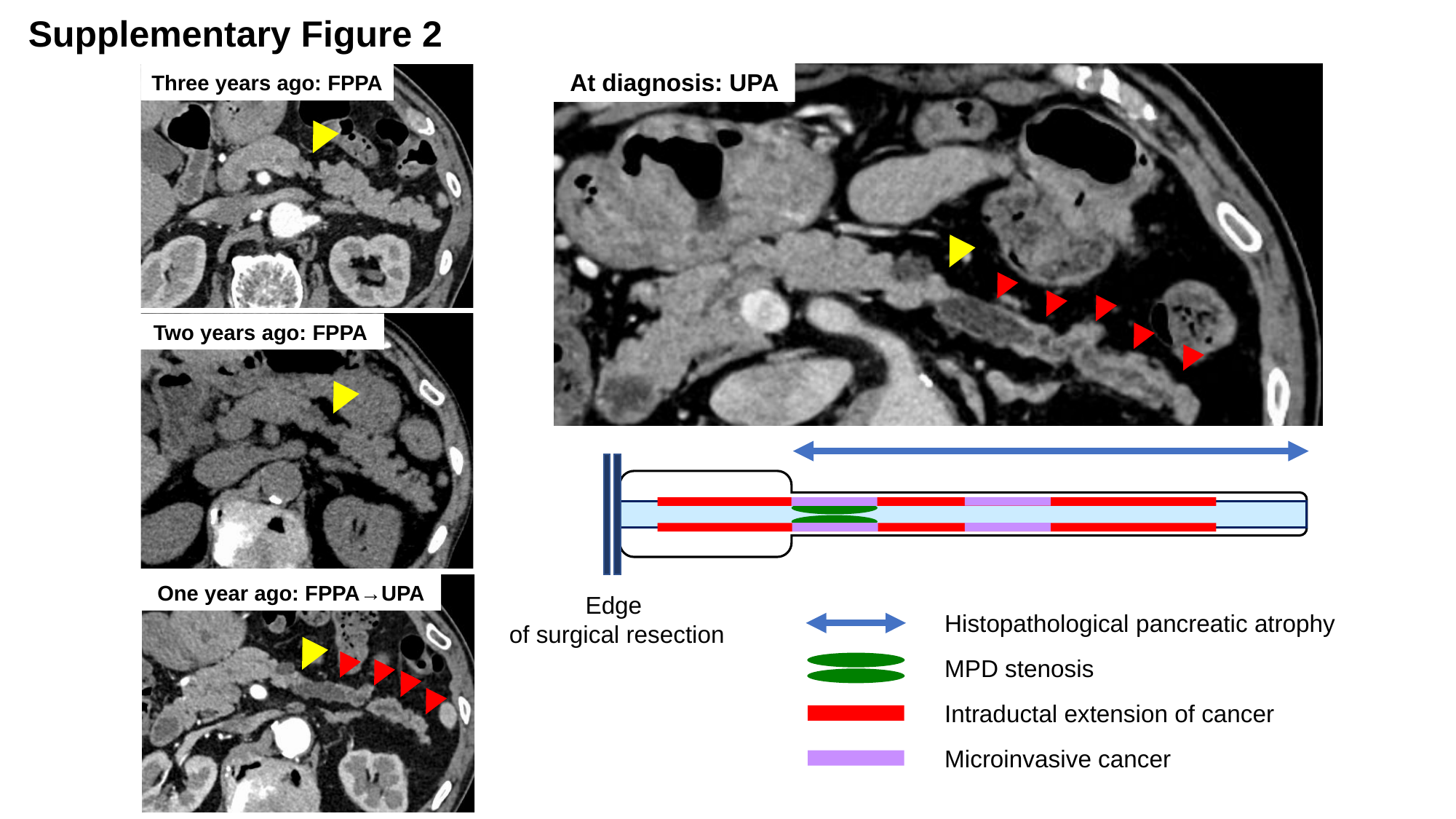

Supplementary Figure 2
At diagnosis: UPA
Three years ago: FPPA
Two years ago: FPPA
One year ago: FPPA→UPA
Edge
of surgical resection
Histopathological pancreatic atrophy
MPD stenosis
Intraductal extension of cancer
Microinvasive cancer
